# The Training of Midwives

**Published:** 1887-02-12

**Authors:** 


### THE TRAINING OF MIDWIVES.

"Matron" writes:—"I have a thoroughly trained and experienced medical and surgical nurse, who is very anxious to take out her certificate for midwifery. I should feel very grateful if any of your readers would inform me at what hospital she could attain her object the most economically, both as regards time and money."

*** "Matron" will find the information she asks for in No. 10 of The Hospital, p. 169.

